# Galectin-3 and Estrogen Receptor Alpha as Prognostic Markers in Prolactinoma: Preliminary Results From a Pilot Study

**DOI:** 10.3389/fendo.2021.684055

**Published:** 2021-07-12

**Authors:** Chiara Bima, Sabrina Chiloiro, Antonella Giampietro, Marco Gessi, Pier Paolo Mattogno, Liverana Lauretti, Carmelo Anile, Guido Rindi, Alfredo Pontecorvi, Laura De Marinis, Antonio Bianchi

**Affiliations:** ^1^ Pituitary Unit, Division of Endocrinology and Metabolism, Fondazione Policlinico Universitario A. Gemelli IRCCS, Università Cattolica del Sacro Cuore, Rome, Italy; ^2^ Department of Medical Science, Division of Endocrinology, Diabetes and Metabolism, A.O.U. “Città della Salute e della Scienza”, Turin, Italy; ^3^ Institute of Pathology, Fondazione Policlinico Universitario A. Gemelli IRCCS, Università Cattolica del Sacro Cuore, Rome, Italy; ^4^ Institute of Neurosurgery, Fondazione Policlinico Universitario A. Gemelli IRCCS, Università Cattolica del Sacro Cuore, Rome, Italy

**Keywords:** prolactinoma, prognosis, estrogen receptor alpha, galectin-3, tumor aggressiveness

## Abstract

**Introduction:**

Prolactin-secreting pituitary tumors (PRL-omas) are generally benign neoplasia. However, a percentage of cases show aggressive behavior. Prognostic markers may allow for the identification of aggressive cases. In this study, we investigated the prognostic role of galectin-3 and the estrogen receptor alpha (ERα), as predictive biomarkers of aggressiveness and poor prognosis.

**Patients and Methods:**

A mono-centric and retrospective study was conducted on consecutive cases of PRL-omas that underwent first line treatment with surgery and were followed-up for at least five years. The immunohistochemical expression of ERα and galectin-3 was investigated in each case.

**Results:**

36 patients were enrolled. Galectin-3 resulted positive in 11 patients (30.6%). The median expression of ERα was 85% (IQR: 37). Among the group of 21 patients who underwent radical surgery (58.3%), recurrence occurred in 12 cases (33.3%). 27 patients were treated post-surgery with a dopamine agonist (DA) (12 for recurrence and 22 for a history of partial surgery). 13 patients (48.1%) were responsive to DA. Six of 11 cases positive for galactin-3 underwent partial surgery (54.5%, p<0.001). Recurrence occurred in all five cases that underwent radical surgery, which were also positive for galectin-3 (p=0.03). Galectin-3 resulted positive in 9 patients resistant to DA treatment (81.1%, p=0.01). ERα expression was lower in tumors positive for galectin-3 (p<0.001), with mitotic activity (p=0.012), with higher Ki67 Li (p<0.001), and in males with post-surgical recurrence (p<0.001).

**Conclusion:**

Galectin-3 and ERα play as markers of aggressiveness and prognosis in PRL-omas and may be tested to identify the aggressive forms of the disease.

## Introduction

Prolactin (PRL)-secreting pituitary adenomas are the most common subtype of pituitary tumors able to secrete hormones (1). The behavior of this neoplasia is very variable, ranging from indolent microadenomas to aggressive, recurrent tumors resistant to DAs. Moreover, in some cases, a malignant transformation was described with the development of metastasis (2, 3). PRL-secreting tumors cover about 30% of pituitary carcinomas (4). The first-line treatment in PRL-secreting pituitary adenomas is medical therapy with dopamine agonists (DAs), however, about 10-15% of these neoplasms show resistance to these treatments, despite indications for surgical removal of PRL-secreting pituitary adenomas being reviewed in recent years.

Until now male gender, invasiveness of para-sellar structures, and histopathological features (as a high proliferative index and an elevated mitotic count) were described as markers of aggressiveness in PRL-secreting tumors. The different gender-related expression of ERα has been hypothesized as involved in the clinical differences of the disease between females and males. However, previous studies and data remain discordant and not conclusive (5–8). Recently, the expression of galectin-3 was suggested as predictive of tumor aggressiveness, but its prognostic role was not yet established (9, 10). Although the molecular and biological factors associated with resistance to DAs have been extensively studied, indications for personalized therapy are not still available.

The study aimed to evaluate the relationship between clinical features, tumor behavior, and the expression of galectin-3 and ERα, in a homogenous and retrospective surgical cohort of patients affected by prolactinoma with long-term follow-up.

## Subjects and Methods

A mono-centric, retrospective, and interventional study was conducted on patients affected by PRL-secreting pituitary adenomas, consecutively enrolled, according to the following inclusion criteria:

1. patients naïve to medical therapy with DA and underwent first line treatment with pituitary neurosurgery, conducted at our institution between 2005 and 2010;
2. patients followed-up for at least five years, at our institution;
3. patients who agreed to participate in the study, signing informed consent;
4. available tumor specimens for interventional IHC evaluations for ERα, SSTR2, and galectin-3.

Exclusion criteria. Patients were excluded from the study if:

1. they underwent medical treatment with DA before pituitary surgery;
2. the histological examination showed a pluri-hormonal IHC positivity of the pituitary adenoma.

### Indications for First Line Surgery

In our institution, also according to Klibanski (11), first-line treatment with pituitary surgery was offered to cases with:

-pituitary apoplexy;
-cystic PRL-omas;
-macro-PRL-omas in patients with a psychiatric condition for which DA is contraindicated;
-after a joint and a multidisciplinary discussion between endocrinologists, neurosurgeons, and patients, concerning risks and benefits, with the final choice based upon the patient’s preference.

### Histopathology

Tumor specimens fixed in formalin and embedded in paraffin (FFPE) were retrieved from the archive of the Department of Pathology, Policlinico A. Gemelli IRCCS. In cases with multiple resections, we studied samples obtained at the time of the first surgery. Tumor specimens were processed according to standard protocols and stained with hematoxylin and eosin (HE). Immunostaining was performed on a Ventana Benchmark XT (Roche Ventana, Darmstadt, Germany) using the following antibodies: anti-PRL (polyclonal, LSBio), anti- Galectin 3 (clone 9C4, Roche), anti-ERα (clone SP1, Ventana), anti-SSTR2 (clone UMB1, abcam), anti-p53 (clone D07, Dako) and anti-Ki67 (MIB1, Dako). The mitotic rate was evaluated by the detection of mitosis on 10 HPF (High power field); the proliferation index was determined as a percentage of nuclear stained cells for KI-67 on at least two foci with the highest number of positive. p53 positivity scored as follows: negative, positive nuclei in < 5% of cells and positive nuclei in ≥ 5% of cells (at least 1500 cells evaluated). The expression of ERα was evaluated as an absolute value.

### Objectives

The main objective of the study was to investigate the prognostic role of galectin-3 and of the estrogen receptor alpha in predicting the outcome of PRL-secreting pituitary tumors.

### Outcomes

All patients underwent first-line neurosurgery. Pituitary surgery was considered subtotal in cases of persistence of hyperprolactinemia and tumor remnant at surgery and/or at MRI performed 3 months after surgery. Radical surgery was defined in cases of recovery of hyperprolactinemia and no evidence of tumor remnant at surgery and MRI performed 3 months after surgery. Recurrence was defined as hormonal and/or radiological disease reactivation during follow-up, in patients who had undergone tumor radical excision and with at least six months of disease-free survival. In fact, in our clinical practice, patients were followed-up according to a strict surveillance program. Hormonal dosage for prolactin and all the other pituitary hormones are conducted one and three months after pituitary surgery and then every six months, for the first five years. Similarly, pituitary MRI is performed six and twelve months after surgery and then annually. After five years of follow-up, cure pituitary hormonal tests are conducted annually and pituitary MR every two years.

During the Al last evaluation available for this study, patients were considered cured if they underwent radical surgery and in absence of recurrence. Patients were considered to carry a persistent disease in cases of subtotal surgery or cases of recurrent disease. Disease status at the end of the study was defined as a measure of outcome. We classified patients as cured or with persistent disease at the end of follow-up.

### Resistance to DA

According to Melmed et al. (12), patients were considered:

-partially resistant to DA in cases of decrease of the tumor size and prolactin levels without normalizing, requiring a higher dose of DA to achieve a complete response;
-complete resistance to DA in cases of failure in obtaining normal prolactin values, and/or failure to reduce tumor size by 50% and/or failure to regain fertility with maximum tolerated doses of DA.

### Statistical Analysis

The normal distribution of continuous variables was evaluated through the Kolmogorov-Smirnov test. Data were presented as median ± interquantile ranges (IQR) for continuous variables and as absolute and relative frequencies for qualitative variables. We performed univariate analysis for the evaluation of the relationship between the considered variables and disease outcome. Fisher exact test and non-parametric tests (Moses, Friedman, Wilcoxon, and Mc-Nemar) were used to compare qualitative and continuous variables, respectively. We performed logistic regression and a survival analysis through Kaplan-Meier curve for the event “recurrence”. Statistical significance was assumed when p<0.05. Data were analyzed using the SSPS Software, version 23.

The study was approved by the local ethics committee of Gemelli Hospital, Catholic University of the Sacred Heart.

## Results

Thirty-six patients entered the study: 13 were women (36.1%) and 23 men (63.9%). The median age of diagnosis was 31 years (IQR: 17). The median duration of follow-up was 96 months (IQR: 56).

Twenty-seven patients carried a macroprolactinoma (75% of cases, median dimension: 14 mm IQR: 9; range: minimum 12 mm, maximum: 35 mm), 7 patients a micro-prolactinoma (19.4% of cases, median dimension: 8 mm IQR: 3, range: minimum 5 mm maximum 9 mm) and 2 patients a giant prolactinoma, defined as larger than 4 cm in diameter (5.6%). Pituitary adenoma was invasive in 14 cases (38.9%): invasion of cavernous sinus occurred in 9 cases (five cases with grade 3 and four cases with grade 4 Knosp score), of dura meter in 2 cases, and sphenoid sinus in 3 cases. All patients underwent first line pituitary surgery. A radical surgical removal was reached in 21 cases and a subtotal removal in 15 cases. Recurrence occurred in 12 out of the 21 cases that underwent radical surgery. Treatment with DA was prescribed in 27 patients (15 for subtotal surgery and 12 for post-surgical recurrence). Mean cabergoline dosage was 1 mg/weekly (minimum: 0.75 mg/weekly maximum 3 mg/weekly). 13 patients (48.1%) were responsive to DA and the remaining 14 (38.9%) were resistant. Three of the 14 DA resistant patients were treated with combination therapy with DA and SSA, reaching the disease control in 2 cases, in terms of normalization of the hyperprolactinemia and the tumor mass. At the end of the study, 9 patients (25%) were considered cured, 16 controlled through post-surgical medical therapy (41.7%), and 12 (33.3%) affected by persistent hyperprolactinemia, despite a high tolerated dose of DA.

### Prognostic Role of Galectin-3 and of the Estrogen Receptor Alpha

Galectin-3 resulted as positive in 11 patients (30.6%) and negative in 25 patients (69.4%). Data on galactin-3 expression in the study population are summarized in **Table 1**. Among the cases positive for galectin-3, 8 were males (72.7%) (p=0.26).

**Table 1:** Galectin-3 expression in study population. Univariate analysis.

	Galectin-3		p-value
	Positive	Negative	
**Gender**			0.367
* Males n, (%)*	8 (72.2%)	15 (60%)	
* Females n, (%)*	3 (27.3%)	10 (40%)	
**Age at PRL-oma diagnosis, median (IQR)**	38 (14.5)	26.5 (13)	0.49
**PRL at diagnosis ng/mL, median (IQR)**	1410 (1530)	663 (3269)	0.76
**KI67 Li, median (IQR)**	2.5 (3.5)	1.5 (2)	0.45
**p53**			0.446
* Negative n, (%)*	7 (26.9%)	19 (73.1%)	
* Positive n, (%)*	4 (40%)	6 (60%)	
**Mitotic count**			0.609
* Negative n, (%)*	5 (45.5%)	11 (44%)	
* Positive n, (%)*	6 (54.5%)	14 (56%)	
**Tumor dimension**			0.147
* Microadenoma n, (%)*	1 (9.1%)	8 (32%)	
* Macroadenoma n, (%)*	10 (90.9%)	17 (68%)	
**Tumor invasiveness**			0.259
* Not-invasive tumors n, (%)*	6 (54.5%)	18 (72%)	
* Invasive tumors n, (%)*	5 (45.5%)	7 (28%)	
**Knosp Score**			0.928
* Grade 0, n, (%)*	9 (32.1%)	19 (67.9%)	
* Grade 3 n, (%)*	1 (25%)	3 (75%)	
* Grade 4 n, (%)*	1 (25%)	3 (75%)	
**Surgical outcome**			0.25
* Radical n, (%)*	5 (45.5%)	16 (64%)	
* Partial n, (%)*	6 (54.%)	9 (36%)	

According to the patients’ prognosis (**Table 2**), we found that radical surgery was reached in 16 out of the 25 cases negative for galactin-3 (64%); instead, a subtotal surgery occurred in 6 out of the 11 cases positive for galactin-3 (54.5%, p<0.001). Recurrence occurred in all five cases that underwent radical surgery and which were positive for galectin-3 (p=0.03). The positivity for galectin-A significantly correlated with the disease persistence (p=0.03). Among the group of 27 patients who underwent DA treatment after surgery, galectin-3 resulted as positive in 9 patients resistant to DA treatment (81.1%) and only in two patients responsive to DA (18.2%, p=0.01), as shown in **Table 3**. In addition, Ki67 was significantly higher in cases resistant to DA (median 3%, IQR: 2, p=0.039), as compared to those that were DA responsive (median 2%, IQR: 2%).

**Table 2:** Predictors of recurrence/progression disease.

	Cured	Persistent disease	p-value
**Number, (%)**	9 (25%)	27 (75%)	
**Gender**			**0.046**
* Males, (%)*	3 (13%)	20 (87%)	
* Females, (%)*	6 (46.2%)	7 (53.8%)	
**Median age at diagnosis (IQR)**	31 (16)	31.5 (12)	0.192
**Median PRL at diagnosis (IQR)**	109 (240)	1310 (2550)	**<0.001**
**Tumor dimension**			0.66
* Microadenoma, (%)*	3 (33.3 %)	6 (66.7%)	
* Macroadenoma, (%)*	6 (22.2%)	21 (77.8%)	
**Tumor invasiveness**			0.108
* Not-invasive, (%)*	8 (33.3%)	16 (66.7%)	
* Invasive, (%)*	1 (8.3%)	11 (91.7%)	
**Knosp Score**			
* Grade 0, n, (%)*	8 (28.6%)	20 (71.4%)	Ref.
* Grade 3 n, (%)*	1 (25%)	3 (75%)	0.882
* Grade 4 n, (%)*	0 (0%)	4 (100%)	0.217
**Ki67, median (IQR)**	1 (1.5)	2 (2.5)	0.154
**p53 expression**			0.667
* Negative, (%)*	6 (23.1%)	20 (76.9%)	
* Positive, (%)*	3 (30%)	7 (70%)	
**Mitosis**			0.245
* Negative, (%)*	7 (35%)	13 (65%)	
* Positive, (%)*	2 (12.5%)	14 (87.5%)	
**Galectin-3**			**0.03**
* Negative, (%)*	9 (36%)	16 (64%)	
* Positive, (%)*	0 (0%)	11 (100%)	
**Estrogen receptor alpha, median (IQR)**	100 (50)	80 (50)	0.965

Univariate analysis. IQR, interquartile range.

**Table 3:** Predictors of responsiveness to DA.

	Dopamine agonist therapy		
	Responsive	Resistant	p-value
**Number, (%)**	13 (48.1%)	14 (38.9%)	Na
**Gender**			0.546
* Males, (%)*	10 (50%)	10 (50%)	
* Females, (%)*	3 (42.9%)	4 (57.1%)	
**Median age at diagnosis (IQR)**	25 (19)	36 (11)	0.183
**Median post-surgical PRL (IQR)**	2552 (3847)	1240 (1700)	0.462
**Tumor dimension**			0.286
* Microadenoma, (%)*	4 (66.7%)	2 (33.3%)	
* Macroadenoma, (%)*	9 (42.9%)	12 (57.1%)	
**Tumor invasiveness**			0.436
* Not-invasive, (%)*	7 (43.8%)	9 (56.3%)	
* Invasive, (%)*	6 (54.5%)	5 (35.7%)	
**Knosp Score**			
* Grade 0, n, (%)*	7 (35%)	13 (65%)	Ref
* Grade 3 n, (%)*	1 (33.3%)	2 (66.7%)	0.295
* Grade 4 n, (%)*	0 (0%)	4 (100%)	**0.03**
**Ki67**	2 (2)	3 (2)	**0.039**
**p53 expression**			0.58
* Negative, (%)*	9 (45%)	11 (55%)	
* Positive, (%)*	4 (57.1%)	3 (42.9%)	
**Mitosis**			0.573
* Negative, (%)*	6 (46.2%)	7 (53.8%)	
* Positive, (%)*	7 (50%)	7 (50%)	
**Galectin-3**			**0.01**
* Negative, (%)*	11 (68.8%)	5 (31.2%)	
* Positive, (%)*	2 (18.2%)	9 (81.8%)	
**Estrogen receptor alpha, median (IQR)**	100 (45)	80 (50)	0.907

Univariate analysis. IQR, interquartile range; DA, dopamine agonist.

**Figure 1:**
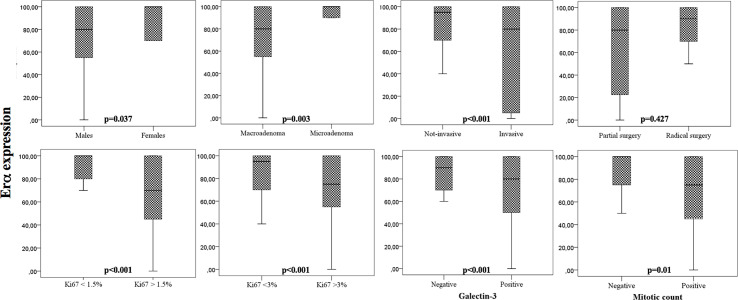
Box-plots that show the expression of ERα according to clinical and pathological parameters. Univariate analysis.

The median expression of ERα was 85% (IQR: 37). ERα expression was significantly lower in males as in females (p=0.037), in macro (p=0.003), and invasive (p<0.001) PRL-secreting tumors (**Figure 1**). In addition, we found that ERα expression correlated with other markers of aggressiveness, resulting in lower in PRL-secreting tumors with mitotic activity (p=0.012), with a Ki67>1.5% (p<0.001) and with Ki67>3% (p<0.001) and in those who expressed the galectin-3 (p<0.001). The ERα expression did not correlate with patients’ age (p=0.514) and PRL values (p=0.584). According to the patients’ prognosis, the expression of estrogen receptor alpha did not correlate with the surgical outcome (p=0.427), recurrence (p=0.245), and responsiveness to DA therapy (p=0.246).

### Survival Analysis

As shown in **Table 4**, the logistic regression showed that the main determinants of disease persistence were a higher value of prolactin at diagnosis > 800 ng/dL (AUC: 0.95; 95%IC: 0.84-1, p=0.03), the male gender (p=0.01) and the positivity of galectin-3 (p=0.03).

**Table 4:** Logistic regression of variables associated to disease persistence and dopamine agonist treatment outcome.

	Disease persistence		Dopamine agonist treatment outcome	
	p-value	HR	p-value	HR
Male gender	0.01	2.5 (1.2-5.6)	Na	Na
Prolactin at diagnosis >800 ng/dL	0.03	3.3 (1.7-6.5)	Na	Na
Knosp score	Na	Na	0.07	Na
Ki67 >1.5%	Na	Na	0.04	1.4(1.1-2.5)
Positive galectin-3	0.03	1.7 (1.2-2.3)	0.03	2.3 (1.1-4.9)

HR, hazard ratio; DA, dopamine agonist.

**Figure 2:**
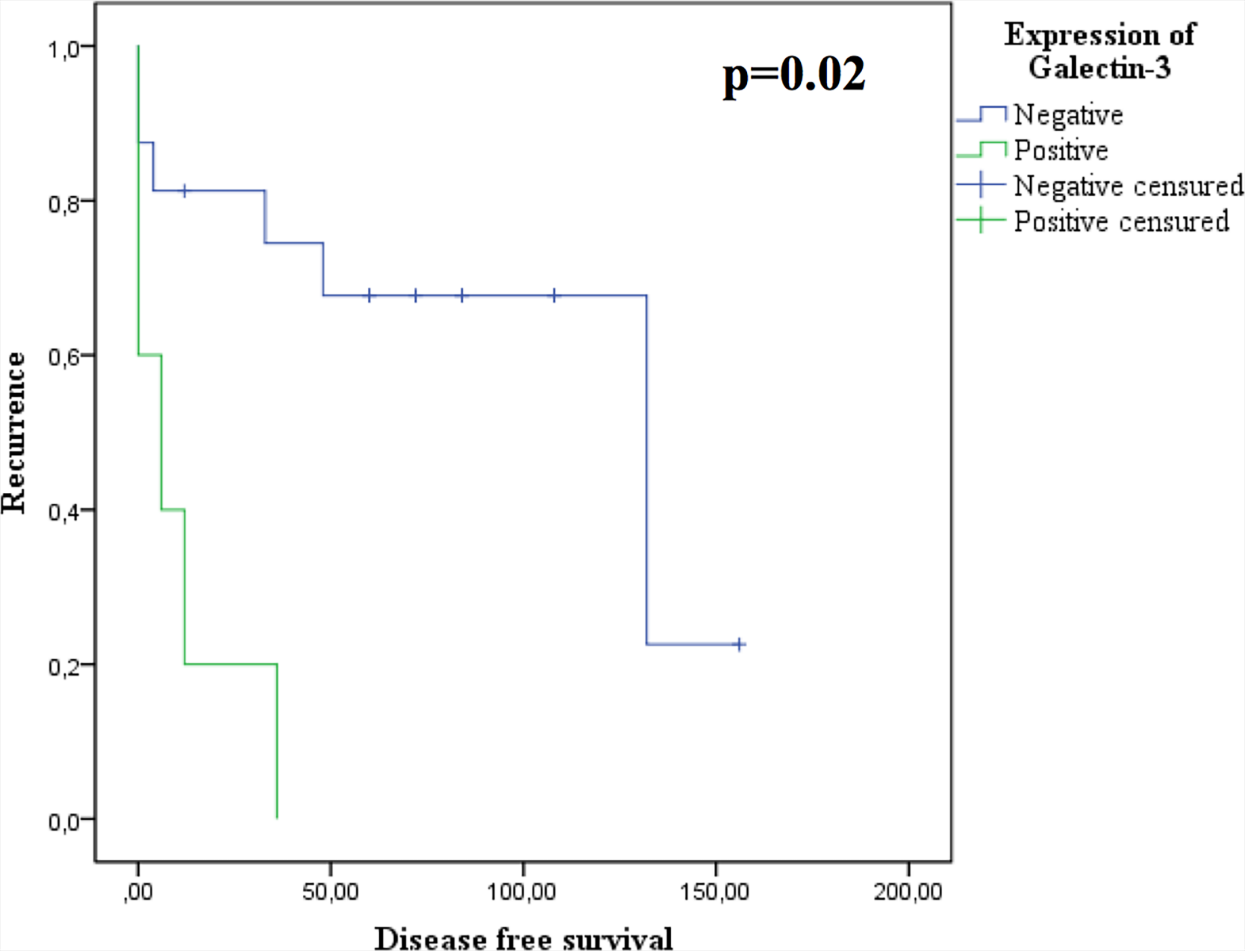
Disease free survival according to galectin-3 expression. Kaplan-Meier curve.

The main determinants of resistance to DA were the Ki67≥ 1.5% (AUC: 0.272 95%IC: 0.07-0.46, p=0.04) and the positivity of galectin-3 (p=0.03). In addition, we found that the expression of galectin-3 correlated with a shorter disease free survival (p=0.02, **Figure 2**).

## Discussion

In this retrospective study, we investigated the prognostic role of galectin-3 and ERα, in predicting aggressiveness, post-surgical outcome, and responsiveness to DA therapy, in patients who underwent first line surgery for PRL-secreting pituitary tumors.

The biological and clinical behavior of PRL-secreting pituitary tumors can be very variable, ranging from cases of micro- PRL-secreting pituitary tumors cured with DA to cases of aggressive tumors characterized by resistance to DAs, recurrence/progression, and at least cases of PRL-secreting pituitary carcinomas (2). Recently, new markers of aggressiveness were suggested in pituitary tumors, as the galectin-3 and the ERα (9–13). Galectin-3 is a β-ganglioside that binds the lectin. The up-regulation of galectin-3 was showed during tumorigenesis in human neoplasms of the thyroid, colon, liver, and brain (12). In the pituitary, galectin-3 is expressed in normal lactotroph and corticotroph cells, resulting in over-expressed pituitary adenomas and carcinomas (13). Zhang et al. suggested that the transcription factors (RUNX1 and RUNX2) of the RUNX family are involved in the regulation of galectin-3, promoting tumor proliferation and progression (14). In particular, Righi et al. (10) demonstrated that invasiveness, high Ki-67 labeling index, and galectin-3 expression were the most important histologic features for assessing the high risk of progression/recurrence, in a cohort of 59 PRL- and 33 ACTH-secreting adenomas. Our results are in line with this previous report but interestingly, suggested that galectin-3 can predict the outcome of PRL-secreting pituitary tumors in terms of post-surgical recurrence, response to DA therapy, and disease-free survival. Few studies have investigated the role of galectin-3 as a marker of aggressiveness in prolactinomas, and our data could help in providing insight into its possible prognostic role in prolactinomas.

Our study also revealed the role of ERα, as a predictor of aggressiveness and prognosis in PRL-secreting pituitary tumors. The expression of ERα in human lactotroph normal and tumoral cells was well established, although in absence of conclusive data on gender difference (5, 6, 15–17). In our study, we proved that the ERα expression is strongly correlated with gender and tumor behavior: males, macro-adenomas, invasive neoplasia and cases with mitotic activity and high Ki-67 (>1.5% and >3%) showed a low expression of ERα. In addition, we found a strong and concordant correlation between the galectin-3 expression and the ERα: cases positive for galectin-3 carried lower ERα.

In a recent study conducted on 89 prolactinomas (7), the low levels of ERα were found to be associated with elevated proliferating markers, high tumor grade, tumor size, invasion, DA resistance, progression after multimodal therapy, and male gender. The inverse correlation between ER expression and markers of proliferation in PRL-secreting tumors might seem surprising, especially considering that estrogens influence cell proliferation and PRL secretion (18, 19). Loss of ERa expression should be considered as a sign of lower differentiation and as a likely indicator of poor prognosis. In parallel, we found that low ERα could predict the recurrence in males, suggesting a prognostic role of these biomarkers, with a gender-related difference. As for consequence, our results supported the hypothesis that the ERα expression may be responsible for the gender differences that are observed in aggressive and malignant lactotroph tumors in men (2). PRL-secreting pituitary tumors are typically larger in males, highly vascularized, more proliferative (7, 20), and less responsive to DA (21).

The higher aggressiveness of PRL-secreting pituitary tumors in males is also well described by the clinico-pathological five-tiered classification of pituitary adenoma proposed by Trouillas et al. (22), as these neoplasms in men are more frequently tumors of higher grade and invasive (2b) with a worse prognosis. Similarly, in the last 2017 WHO Classification of the Tumours of the Pituitary Gland, prolactinomas in male patients were identified as one of the variants of pituitary tumors with a “high risk for recurrence” (23).

Other markers of aggressiveness in our series were the higher value of prolactin at diagnosis and the higher value of Ki67 that may predict the resistance to DA. As shown in previous papers (24), this study confirms that Ki67 is a prognostic biomarker in pituitary adenomas. Moreover, in this cohort, we did not identify a prognostic role of p53 in predicting the outcome of pituitary adenomas, as demonstrated by several authors. For these reasons, according to the not conclusive data on p53 in adeno-pituitary tumors, the p53 was removed as a prognostic marker from the 2017 WHO Classification of the Tumours of the Pituitary Gland (23).

The main limitations of our study are the small sample size and its retrospective design. However, this study described a monocentric series of patients affected by PRL-secreting pituitary tumors naïve to DA therapy and underwent first line treatment with pituitary surgery. At our institution, over the years, trans-sphenoidal pituitary surgery has been considered an important part of patient counseling in deciding upon an initial treatment, according to the low morbidity of this procedure, if performed by an experienced neurosurgeon (25). In particular, at our center, pituitary surgery was proposed as first line treatment choice in cases with pituitary apoplexy, cystic PRL-secreting pituitary tumors, macro-PRL-oma in patients with a psychiatric condition for which dopamine agonists were contraindicated, or after a joint and multidisciplinary discussion between endocrinologists, neurosurgeons, and patients, concerning risks and benefits, with the final choice based upon the patient’s preference (11). Another limitation of our study was the absence of analysis of the correlation between the expression of the different subtypes of the somatostatin receptors (SSTR) and the responsiveness to SSA. In this series, only three patients were treated with SSAs. Two out of these three patients reached the disease control, despite the absence of expression of the subtype 2 of SSTR (SSTR2). In this series, the SSTR2 was detected only in three patients. Our data are concordant with experimental data, demonstrating that SSTR5, unlike SSTR2, is the most important receptor for the regulation of PRL secretion (26, 27). However, some clinical cases reported that patients with DA-resistant prolactinomas were successfully treated with octreotide LAR and cabergoline combination treatment (28–30). The ability of SSA in reducing the prolactin secretion and inducing a shrinkage may be due to the affinity of Octreotide Lar and Lanreotide Lar in binding the subtype 5 of the SSTR, despite a lower affinity as compared to the SSTR2. In this context, nuclear imaging techniques can be useful for predicting responsiveness to SSA also in PRL-secreting pituitary tumors (28, 29).

In conclusion, although our results should be confirmed by a larger cohort and prospective studies, our results suggest that ERα and galectin-3 are markers of aggressiveness and prognosis in PRL-secreting pituitary tumors and may be tested for identifying aggressive forms of the disease, in the field of personalized therapy, with a multidisciplinary approach.

## Data Availability Statement

The raw data supporting the conclusions of this article will be made available by the authors, without undue reservation.

## Ethics Statement

The studies involving human participants were reviewed and approved by Ethics committee of Fondazione Policlinico Gemelli, Rome. The patients/participants provided their written informed consent to participate in this study.

## Author Contributions

CB and SC wrote the manuscript. CB performed the conceptualization, data curation, and data collection. SC performed the conceptualization and formal analysis. MG developed the methodology and conducted the histological investigation. AG, PM, LL, CA, GR, AP, LM, and AB conducted the validation of the research outputs. AP, LM, and AB performed the supervision of the research activities. All authors contributed to the article and approved the submitted version.

## Conflict of Interest

The authors declare that the research was conducted in the absence of any commercial or financial relationships that could be construed as a potential conflict of interest.
