# Supplementary material for: Galectin-3 and Estrogen Receptor Alpha as Prognostic Markers in Prolactinoma: Preliminary Results From a Pilot Study
Source: Front Endocrinol (Lausanne). 2021 Jul 12;12:684055. doi: 10.3389/fendo.2021.684055 (PMC8312245; doi:10.3389/fendo.2021.684055)
Supplement: Supplementary file 1 [file Table_1.docx]

**Table 1. Galectin-3 expression in study population. Univariate analysis.**

|  | **Galectin-3** | | **p-value** |
| --- | --- | --- | --- |
|  | **Positive** | **Negative** |  |
| **Gender**  *Males n, (%)*  *Females n, (%)* | 8 (72.2%)  3 (27.3%) | 15 (60%)  10 (40%) | 0.367 |
| **Age at PRL-oma diagnosis, median (IQR)** | 38 (14.5) | 26.5 (13) | 0.49 |
| **PRL at diagnosis ng/mL, median (IQR)** | 1410 (1530) | 663 (3269) | 0.76 |
| **KI67 Li, median (IQR)** | 2.5 (3.5) | 1.5 (2) | 0.45 |
| **p53**  *Negative n, (%)*  *Positive n, (%)* | 7 (26.9%)  4 (40%) | 19 (73.1%)  6 (60%) | 0.446 |
| **Mitotic count**  *Negative n, (%)*  *Positive n, (%)* | 5 (45.5%)  6 (54.5%) | 11 (44%)  14 (56%) | 0.609 |
| **Tumor dimension**  *Microadenoma n, (%)*  *Macroadenoma n, (%)* | 1 (9.1%)  10 (90.9%) | 8 (32%)  17 (68%) | 0.147 |
| **Tumor invasiveness**  *Not-invasive tumors n, (%)*  *Invasive tumors n, (%)* | 6 (54.5%)  5 (45.5%) | 18 (72%)  7 (28%) | 0.259 |
| **Knosp Score**  *Grade 0, n, (%)*  *Grade 3 n, (%)*  *Grade 4 n, (%)* | 9 (32.1%)  1 (25%)  1 (25%) | 19 (67.9%)  3 (75%)  3 (75%) | 0.928 |
| **Surgical outcome**  *Radical n, (%)*  *Partial n, (%)* | 5 (45.5%)  6 (54.%) | 16 (64%)  9 (36%) | 0.25 |

**Table 2. Predictors of recurrence/progression disease. Univariate analysis.**

|  | **Cured** | **Persistent disease** | **p-value** |
| --- | --- | --- | --- |
| **Number, (%)** | 9 (25%) | 27 (75%) |  |
| **Gender**  *Males, (%)*  *Females, (%)* | 3 (13%)  6 (46.2%) | 20 (87%)  7 (53.8%) | **0.046** |
| **Median age at diagnosis (IQR)** | 31 (16) | 31.5 (12) | 0.192 |
| **Median PRL at diagnosis (IQR)** | 109 (240) | 1310 (2550) | **<0.001** |
| **Tumor dimension**  *Microadenoma, (%)*  *Macroadenoma, (%)* | 3 (33.3 %)  6 (22.2%) | 6 (66.7%)  21 (77.8%) | 0.66 |
| **Tumor invasiveness**  *Not-invasive, (%)*  *Invasive, (%)* | 8 (33.3%)  1 (8.3%) | 16 (66.7%)  11 (91.7%) | 0.108 |
| **Knosp Score**  *Grade 0, n, (%)*  *Grade 3 n, (%)*  *Grade 4 n, (%)* | 8 (28.6%)  1 (25%)  0 (0%) | 20 (71.4%)  3 (75%)  4 (100%) | Ref.  0.882  0.217 |
| **Ki67, median (IQR)** | 1 (1.5) | 2 (2.5) | 0.154 |
| **p53 expression**  *Negative, (%)*  *Positive, (%)* | 6 (23.1%)  3 (30%) | 20 (76.9%)  7 (70%) | 0.667 |
| **Mitosis**  *Negative, (%)*  *Positive, (%)* | 7 (35%)  2 (12.5%) | 13 (65%)  14 (87.5%) | 0.245 |
| **Galectin-3**  *Negative, (%)*  *Positive, (%)* | 9 (36%)  0 (0%) | 16 (64%)  11 (100%) | **0.03** |
| **Estrogen receptor alpha, median (IQR)** | 100 (50) | 80 (50) | 0.965 |

**Table 3. Predictors of responsiveness to DA. Univariate analysis.**

|  | **Dopamine agonist therapy** | | |
| --- | --- | --- | --- |
|  | **Responsive** | **Resistant** | **p-value** |
| **Number, (%)** | 13 (48.1%) | 14 (38.9%) | Na |
| **Gender**  *Males, (%)*  *Females, (%)* | 10 (50%)  3 (42.9%) | 10 (50%)  4 (57.1%) | 0.546 |
| **Median age at diagnosis (IQR)** | 25 (19) | 36 (11) | 0.183 |
| **Median post-surgical PRL (IQR)** | 2552 (3847) | 1240 (1700) | 0.462 |
| **Tumor dimension**  *Microadenoma, (%)*  *Macroadenoma, (%)* | 4 (66.7%)  9 (42.9%) | 2 (33.3%)  12 (57.1%) | 0.286 |
| **Tumor invasiveness**  *Not-invasive, (%)*  *Invasive, (%)* | 7 (43.8%)  6 (54.5%) | 9 (56.3%)  5 (35.7%) | 0.436 |
| **Knosp Score**  *Grade 0, n, (%)*  *Grade 3 n, (%)*  *Grade 4 n, (%)* | 7 (35%)  1 (33.3%)  0 (0%) | 13 (65%)  2 (66.7%)  4 (100%) | Ref  0.295  **0.03** |
| **Ki67** | 2 (2) | 3 (2) | **0.039** |
| **p53 expression**  *Negative, (%)*  *Positive, (%)* | 9 (45%)  4 (57.1%) | 11 (55%)  3 (42.9%) | 0.58 |
| **Mitosis**  *Negative, (%)*  *Positive, (%)* | 6 (46.2%)  7 (50%) | 7 (53.8%)  7 (50%) | 0.573 |
| **Galectin-3**  *Negative, (%)*  *Positive, (%)* | 11 (68.8%)  2 (18.2%) | 5 (31.2%)  9 (81.8%) | **0.01** |
| **Estrogen receptor alpha, median (IQR)** | 100 (45) | 80 (50) | 0.907 |

|  | Disease persistence | | Dopamine agonist treatment outcome | |
| --- | --- | --- | --- | --- |
|  | p-value | HR | p-value | HR |
| Male gender | 0.01 | 2.5 (1.2-5.6) | Na | Na |
| Prolactin at diagnosis >800 ng/dL | 0.03 | 3.3 (1.7-6.5) | Na | Na |
| Knosp score | Na | Na | 0.07 | Na |
| Ki67 >1.5% | Na | Na | 0.04 | 1.4(1.1-2.5) |
| Positive galectin-3 | 0.03 | 1.7 (1.2-2.3) | 0.03 | 2.3 (1.1-4.9) |

Table 4. Logistic regression of variables associated to disease persistence and dopamine agonist treatment outcome. HR: hazard ratio
